# Effects of Passive or Active Recovery Regimes Applied During Long-Term Interval Training on Physical Fitness in Healthy Trained and Untrained Individuals: A Systematic Review

**DOI:** 10.1186/s40798-024-00673-0

**Published:** 2024-03-05

**Authors:** Hassane Zouhal, Abderraouf Ben Abderrahman, Ayyappan Jayavel, Anthony C. Hackney, Ismail Laher, Ayoub Saeidi, Fatma Rhibi, Urs Granacher

**Affiliations:** 1 M2S (Laboratoire Mouvement, Sport, Santé) - EA 1274, Univ Rennes, 35000 Rennes, France; 2 Institut International des Sciences du Sport (2I2S), 35850 Irodouër, France; 3 Higher Institute of Sport and Physical Education of Ksar-Said, University of Manouba, Manouba, Tunisia; 4 Tunisian Research Laboratory “Sports Performance Optimization”, National Center of Medicine and Science in Sports (CNMSS) LR09SEP01, Tunis, Tunisia; 5 SRM College of Physiotherapy, SRM Institute of Science and Technology, SRM Nagar, Kattankulathur, Kanchipuram, Chennai, TN 603203 India; 6 Department of Exercise and Sport Science, University of North Carolina, Chapel Hill, NC USA; 7 Department of Anesthesiology, Pharmacology and Therapeutics, The University of British Columbia, Vancouver, Canada; 8 Department of Physical Education and Sport Sciences, Faculty of Humanities and Social Sciences, University of Kurdistan, Sanandaj, 66177-15175 Kurdistan Iran; 9 Department of Sport and Sport Science, Exercise and Human Movement Science, University of Freiburg, Freiburg, Germany

**Keywords:** Recovery mode, Interval training, Physiological parameters, Performances, Healthy adults

## Abstract

**Background:**

Intermittent exercise programs characterized through intensive exercise bouts alternated with passive or active recovery (i.e., interval training), have been proven to enhance measures of cardiorespiratory fitness. However, it is unresolved which recovery type (active or passive) applied during interval training results in larger performance improvements.

**Objectives:**

This systematic review aimed to summarize recent evidence on the effects of passive or active recovery following long-term interval exercise training on measures of physical fitness and physiological adaptations in healthy trained and untrained individuals. The study protocol was registered in the Open Science Framework (OSF) platform (https://doi.org/10.17605/OSF.IO/9BUEY).

**Methods:**

We searched nine databases including the grey literature (Academic Search Elite, CINAHL, ERIC, Open Access Theses and Dissertations, Open Dissertations, PsycINFO, PubMed/MEDLINE, Scopus, and SPORTDiscus) from inception until February 2023. Key terms as high-intensity interval training, recovery mode, passive or active recover were used. A systematic review rather than a meta-analysis was performed, as a large number of outcome parameters would have produced substantial heterogeneity.

**Results:**

After screening titles, abstracts, and full texts, 24 studies were eligible for inclusion in our final analysis. Thirteen studies examined the effects of interval training interspersed with passive recovery regimes on physical fitness and physiological responses in trained (6 studies) and untrained (7 studies) individuals. Eleven out of 13 studies reported significant improvements in physical fitness (e.g., maximal aerobic velocity (MAV), Yo-Yo running test, jump performance) and physiological parameters (e.g., maximal oxygen uptake [VO_2max_], lactate threshold, blood pressure) in trained (effect sizes from single studies: 0.13 < Cohen’s *d* < 3.27, small to very large) and untrained individuals (effect sizes: 0.17 < *d* < 4.19, small to very large) despite the type of interval training or exercise dosage (frequency, intensity, time, type). Two studies were identified that examined the effects of passive recovery applied during interval training in young female basketball (15.1 ± 1.1 years) and male soccer players (14.2 ± 0.5 years). Both studies showed positive effects of passive recovery on VO_2max_, countermovement jump performance, and the Yo-Yo running test. Eleven studies examined the effects of interval training interspersed with active recovery methods on physical fitness and physiological parameters in trained (6 studies) and untrained individuals (5 studies). Despite the type of interval training or exercise dosage, nine out of eleven studies reported significant increases in measures of physical fitness (e.g., MAV) and physiological parameters (e.g., VO_2max_, blood pressures) in trained (effect sizes from single studies: 0.13 < *d *< 1.29, small to very large) and untrained individuals (effect sizes: 0.19 < *d* < 3.29, small to very large). There was no study available that examined the effects of active recovery on physical fitness and physiological responses in youth.

**Conclusions:**

The results of this systematic review show that interval training interspersed with active or passive recovery regimes have the potential to improve measures of physical fitness and physiology outcomes in trained and untrained adults and trained youth. That is, the applied recovery type seems not to affect the outcomes. Nonetheless, more research is needed on the effects of recovery type on measures of physical fitness and physiological adaptations in youth.

## Key Points

- Endurance training in the form of interval training is known to increase physical fitness (e.g., MAV) and physiological parameters (e.g., VO_2max_, blood pressure) in both trained and untrained individuals, irrespective of age and sex.
- This review revealed that interval training improves measures of physical fitness (e.g., MAV) and physiological parameters (e.g., VO_2max_, blood pressure) similarly in trained and untrained adults and trained youth, regardless of the type of exercise and the exercise dosage.
- The applied recovery type (active or passive) during interval training seems not to affect the training-induced outcomes, irrespective of the training status (trained versus untrained) and sex.

## Background

Maximal oxygen uptake (VO_2max_) is an important physiological determinant of sports performance particularly in metabolically demanding sports such as middle and long-distance running, cycling, rowing, cross-country skiing, etc. [1–5]. Training-induced improvements in VO_2max_ depend on factors, such as age, sex, and training status [6–9]. Accordingly, training protocols with the goal to improve VO_2max_ should be individualized according to the needs of the athlete [10–13]. Nevertheless, the available literature provides some guidance on the programming of exercise protocols to enhance VO_2max_. For instance, for the training modalities exercise frequency, intensity, time, and type of exercise (FITT-principle), there is evidence that training at or near an individual's VO_2max_ may be an ideal stimulus to increase VO_2max_ [3, 14]. Regarding the type of training, it seems that intermittent exercise protocols consisting of intensive exercise bouts alternated with passive or active recovery regimes can improve cardiorespiratory fitness [4–6, 15–18].

Interestingly, high-intensity interval training (HIIT) has not only been applied in sub-elite and elite athletes to enhance their aerobic capacity but also in recreational athletes and even in patients (e.g., individuals with obesity, diabetes, etc.) [19–23]. Despite its widespread use, there is no common definition of HIIT; in the context of performance, HIIT can be defined as intermittent bouts of exercise realized at high-intensity, and in the context of health, HIIT can be characterized as intermittent exercise performed at low or moderate intensity [24]. As such, the dosage of HIIT protocols varies greatly and differs in exercise intensity, duration of intervals, number of repetitions, recovery types, work-to-rest ratio, and rest time between interval bouts [10, 11]. The application of HIIT protocols is particularly popular in intermittent sports, such as team sports (e.g., soccer) or racket sports (e.g., tennis), to improve measures of physical fitness [25, 26]. Physiological adaptations following HIIT are based on complex molecular (e.g., the expression of PGC-1α mRNA) and cellular mechanisms (e.g., mitochondrial density and biogenesis) [27, 28].

During the performance of HIIT sessions, immediate post-exercise recovery (i.e., after each repetition or interval), represents an important restorative process (e.g., physiological, psychological) that impacts on the magnitude of the training-induced physiological adaptations [29, 30]. Both the type of recovery (active, passive) as well as the recovery time influence maximal performance during each interval and the overall physiological stress [31, 32].

Active recovery at low-to-moderate intensities during HIIT may enable larger adaptive potential during the next HIIT exercise bout than passive recovery, but the experimental data to support this claim are inconclusive [33]. While some studies report a greater magnitude of adaptation with active recovery regimes [4, 5, 34, 35], other studies indicate that the type of recovery does not have an impact on training-induced adaptations [36]. A recent systematic review focused only on acute physiological, perceptual, and performance effects of recovery mode applied between repeated-sprints during running and cycling protocols reported that passive recovery reduced physiological and perceptual demands and reduced loss of performance compared to active recovery in repeated-sprints running, with limited data on cycling studies [37]. In contrast, another systematic review on the effects of recovery mode on performance limited to mean and peak power, time to exhaustion, and distance covered during an interval exercise session only indicated that passive recovery aids in maintaining performance during interval exercise [38].

Accordingly, it seems timely to systematically summarize the literature to identify whether interval training with active or passive recovery shows larger adaptive potential after a long-term exercise training. The objective of this systematic review was to gather recent evidence on the effects of the recovery type *(passive or active)* applied during long-term interval training on measures of physical fitness and physiological adaptations in healthy trained and untrained youth and adult individuals.

## Methods

### Procedures

This systematic review was conducted in accordance with the Preferred Reporting Items for Systematic Reviews and Meta-Analyses (PRISMA) Statement [39]. The study protocol was registered in the Open Science Framework (OSF) platform (https://doi.org/10.17605/OSF.IO/9BUEY).

The PICOS approach (Population, Intervention, Comparator, Outcomes, Study design) was followed to identify inclusion criteria (Table 1). Only randomized controlled trials and controlled trials that examined the effects of passive or active recovery during long-term interval training, for at least 3 weeks, on measures of physical fitness and physiological adaptations in trained and untrained youth and adult individuals were eligible for inclusion. The following criteria were a priori defined for studies to be eligible for inclusion in this systematic review article: (1) published in peer-reviewed journals; (2) included healthy trained and untrained individuals, irrespective of age; (3) used validated methods of exercise training quantification; (4) used training programs with passive or active recovery types; (5) used physical fitness tests (e.g., Léger & Boucher test, VAMEVAL) and physiological tests (e.g., VO_2max_); (6) were written in English, and (7) exercise interventions lasted a minimum of 3 weeks.

**Table 1:** Inclusion criteria according to the PICOS approach

PICOS components	Details
Population	Healthy trained and untrained humans aged > 14 years old
Intervention	Interval exercise training using passive or active recovery types during exercise for at least 3 weeks
Comparator	Active and/or passive controls
Outcomes	Measures of physical fitness (e.g., aerobic performance, jump performance etc.), physiological adaptations (e.g., maximal oxygen uptake [VO_2max_], maximal aerobic speed [MAS], lactate threshold, running economy etc.)
Study Design	nRCTs, nRnCTs and RCTs

*nRCT* Non-randomized controlled trial, *nRnCT* Non-randomized uncontrolled trial, *RCT* Randomized controlled trial

Studies were excluded if they (1) did not meet the minimum requirements of an experimental study design (e.g., case reports), (2) did not meet the minimum requirements regarding training design (e.g., lack of information on training methodology or testing sessions), (3) were not written in English, and (4) involved individuals with clinical concerns. Published review articles were also excluded from our analysis.

In the current review, “trained individuals” refers to athletes who exercised at least three times per week, and “untrained” refers to individuals who did not meet the World Health Organization minimum activity guidelines of 60 min per day (youth) and 150 min per week (adults) [40].

### Literature Search Strategy

We searched nine databases including the grey literature (Academic Search Elite, CINAHL, ERIC, Open Access Theses and Dissertations, Open Dissertations, PsycINFO, PubMed/MEDLINE, Scopus, and SPORTDiscus) from inception until February 2023.

The following key terms (and synonyms searched for using the MeSH term database) were included and combined using the operators “AND”, “OR”,: [(exercise OR training OR “exercise training” OR “interval exercise” OR “intermittent exercise” OR “interval training” OR “intermittent training” OR “high-intensity interval exercise” OR “high-intensity interval training”) AND (recovery OR “recovery mode” OR “passive recovery” OR “active recovery”) AND (“physical fitness” OR “physiological adaptation*” OR “physiological response*”)].

In addition, the reference lists and citations (Google Scholar) of included studies were explored further to detect additional related studies. Since the scope of this systematic review article is large in terms of outcome measures (e.g., physical fitness, physiological adaptations), we performed a systematic review rather than a meta-analysis, as a large number of outcome parameters would have produced substantial heterogeneity.

### Study Selection

The final screening was done by two investigators (FR and AJ) based on the relevance of the inclusion and exclusion criteria and the identified items for assessing the effects of passive or active recovery after long-term interval training (at least 3 weeks) on measures of physical fitness and physiological adaptations in trained and untrained individuals using PICOS criteria. If the title of the article was potentially relevant, the manuscript was screened at the abstract level. When abstracts indicated potential inclusion, full texts were reviewed. A third-party consensus meeting was held with another author (HZ) if the two reviewers were not able to reach a consensus.

### Quality and Risk of Bias and Assessment

The methodological quality of the included studies was assessed using the Physiotherapy Evidence Database (PEDro) scale (http://www.pedro.fhs.usyd.edu.au), which has good reliability and validity [39]. The PEDro scale has eleven possible points that examine external validity (criterion 1) and internal validity (criteria 2–9) of controlled trials and whether there is sufficient statistical information for interpreting the results (criteria 10–11). A cut-off score of six on the PEDro scale was used to differentiate between low and high methodological quality [41]. Two independent researchers (FR and ABA) assessed the quality of the studies, and if any unambiguity arose, a third researcher (HZ) was contacted to reach a unanimous decision.

### Statistics

The percent change (Δ%) was calculated (if not available in the study) for each study to evaluate the magnitude of the effects using the following equation:

$$\mathrm{\Delta \%}=\left(Mpost-Mpre\right)/Mpre\times 100$$

where Mpost represents the mean value after intervention and Mpre the baseline mean value.

Effect sizes (ES) were computed for single studies but were not aggregated across studies to present standardized effects of exercise training on the outcome variables (e.g., physical fitness and physiological adaptations). As an ES measure, we consistently used Cohen’s d [42] by dividing the raw ES (difference in means) by the pooled standard deviations:

$$ES=(\mathrm{Cohen{\prime}}\mathrm{s d})=({\text{M}}1-{\text{M}}2)/\mathrm{SD pooled})$$

Values for ES were defined as trivial (< 0.2), small (0.2–0.6), moderate (0.6–1.2), large (1.2–2.0), and very large > 2.0 [43]. Results for each outcome variable are presented with several observations (N), Δ%, and ES. The data analysis was processed using SigmaStat 3.5 software (Systat, Inc, USA). The ES and Δ% were analyzed in studies where sufficient data were available. A significant difference was indicated when the 95% confidence interval (CI) of the ES did not overlap zero.

## Results

### Study Selection

**Fig. 1:**
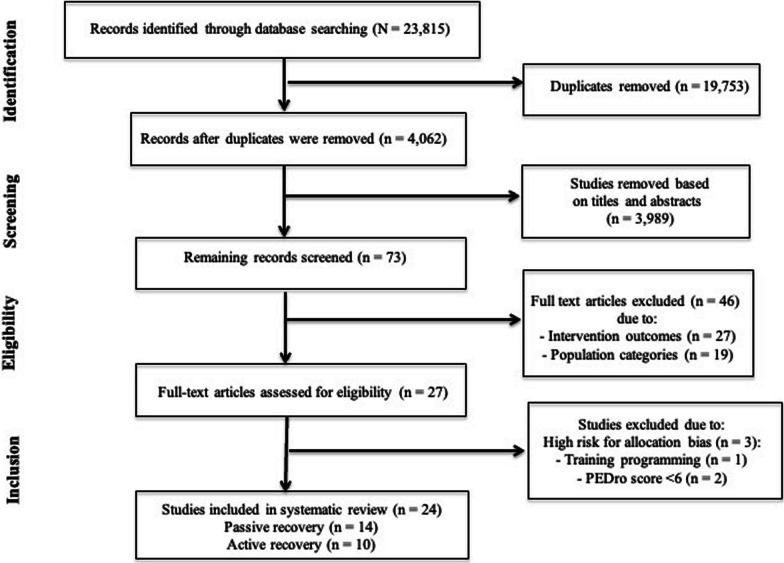
Selection process for research articles (*N* = 24) included in this systematic review [39]

We identified 23,815 studies related to the effects of long-term interval training on physical fitness and physiological parameters in healthy trained and untrained individuals (Fig. 1). After the screening of titles, abstracts, and full texts, 24 studies were eligible to be included in our final analysis. The characteristics of the included studies are summarized in Table 2. A total of 501 individuals participated in the interval training programs with active recovery and 229 in interval training programs with passive recovery regimes. Participants’ age ranged from 14 to 48 years.

**Table 2:** Characteristics of the studies that examined the effects of interval training with active or passive recovery on measures of physical fitness and physiological adaptations in trained and untrained individuals

Study	PEDro score	Country	Sample size/sex	Physical activity level/sport	Age, years (mean ± SD or range)	Recovery type	Training volume	Training intensity	Intervention duration (weeks)	Session duration (min)	Training frequency
Buckley et al. [54]	7	Canada	28 healthy females	Active	24.7 ± 5.4	Passive	Row-HIIT and MM-HIIT group performed 60 s of all-out intensity rowing followed by 3 min of rest (passive recovery) for a total of 6 rounds. High-volume	All out intensity; 9/10 or 10/10 in RPE scale	6 weeks	24	3 times/week
Kong et al. [53]	7	Macau	18 overweight and obese females	Inactive	18–30	Passive	60 repetitions of high-intensity interval exercise (8 s cycling and 12 s passive recovery) on a cycle ergometer for 20 min. High-volume	90% VO_2max_	5 weeks	20 min	4 session/week
Aschendorf et al. [49]	7	Germany	24 females	Basketball	15.1 ± 1.1	Passive	Two different types of HIIT: Session A 4 × 4 HIIT with 3 min of recovery; Session B 2 × 30 s HIIT with 15 s recovery. High and low volume	90–95%VO_2max_	5 weeks	25 min	3 times/week
Wiewelhove et al. [26]	7	Germany	26 males	Soccer, handball, basketball, tennis	23.5 ± 2.5	Passive	Straight-line runs at 95–100%vVO_2_ with 2–4 min of rest. Low-volume	90–100%	4 weeks	40 min	3 times/week
Jabbour et al. [52]	6	Qatar	10 males and 20 females	Inactive	18–71	Passive	6 repetitions of SCT intervals with 2 min of passive recovery between each repetition. Low-volume	all out SCT	6 weeks	15 min	3 times/week
Fransson et al. [47]	7	Sweden	39 males	Soccer	21.1 ± 2.4	Passive	The drill was performed as a time trial drill in 30-s intervals separated by 150 s of passive recovery. The number of exercise intervals was 6 during the first intervention week, 8 during the second and third weeks, and 10 during the fourth week. High-volume	maximal speed drills	4 weeks	30 min	3 times/week
Menz et al. [50]	7	Austria	15 participants (female (*n *= 11) and male (*n *= 4) (age 25.6 ± 2.6 years)	Running, cycling, fitness, ball sports and alpine sports	25.6 ± 2.6	Passive	Running HIIT (3–4 sets; 8 × 20 s, 10 s rest; set rest: 5 min)/Functional HIIT executed with their own body weight (3–4 sets; 8 × 20 s, 10 s rest; set rest: 5 min). Low-volume	90–100% HR_max_	4 weeks	15–20 min	3 times/week
Evangelista et al. [55]	7	Brazil	25 males and females	Active	28.3 ± 6.8	Passive	20 sets of 30 s all-out exercise and 30 s of passive recovery between sets. High-volume	All out exercise	6 weeks	20 min	3 times/week
Alizadeh and Safarzade [51]	7	Spain	20 adolescent boys	Active	18.0 ± 1.5	Passive	3 × 10 repetitions of 30 s of aerobic exercises all out (cycling, rowing, and running) interspersed by 30 s of rest. High-volume	90% HR_max_	6 weeks	10–20 min	3 times/week
Moro et al. [56]	7	Italy	20 healthy young adults	Inactive	22.1 ± 1.9	Passive	HIIRT: first set of 6 repetitions at 80% 1RM followed by a 20″ rest; then, subjects were asked to lift the same weight until failure (habitually 2 or 3 repetitions) followed by another 20″ rest period with repetitions to fatigue. Low-volume	80% of 1RM	6 weeks	43 min (including warm up and cool down)	3 times/week
Arslan et al. [48]	6	Turkey	20 males	Soccer	14.2 ± 0.5	Passive	Intermittent running at 90–95% of players’ VIFT for 15 s (around the pitch), followed by 15 s of passive recovery. Low-volume	90–95% VIFT	5 weeks	5–10 min	2 times/week
Martínez-Rodríguez et al. [57]	7	Spain	14 females	Active	27 ± 6	Passive	3 × 10 repetitions of 30 s of aerobic exercises all out (cycling, rowing, and running) interspersed by 30 s of rest. High-volume	All out training	8 weeks	40 min	3 times/week
Ben Abderrahman et al. [4]	7	Tunisia	9 males	Physical education students	20.4 ± 0.6	Passive	2 set x (8 rep x 30sIE) with 5 min recovery. High-volume	100% MAV	7 weeks	45 (including warm up and cool down)	3 times/week
Martins et al. [45]	7	Norway	30 females and 16 males	Sedentary	34.4 ± 8.8	Active	8 s of sprinting (during which participants worked as hard as possible) and 12 s of recovery phase. Low-volume	Exercise: all out	12 weeks	20 min	3 times/week
Ben Abderrahman et al. [4]	7	Tunisia	9 males	Physical education students	20.9 ± 0.8	Active	2 set x (8 rep x 30sIE) 100% MAV with 5 min recovery at 50% MAV. High-volume	Exercise: 100% MAV. recovery: 50%	7 weeks	45 min (including warm up and cool down)	3 times/week
Heydari et al. [63]	7	Australia	34 males	Inactive	24.4 ± 4.7	Active	8 s sprint and 12 s recovery, and a 5-min cool-down. During recovery, the cadence was reduced to 40 rpm with no change in resistance. Participants kept their exercise intensity at a level with 80–90% MHR. Low-volume	Exercise: all out recovery: 40 rpm	12 weeks	20 min	3 times/week
Trapp et al. [46]	7	Australia	34 females	Inactive	22.4 ± 0.7	Active	Each subject performed 8 s of sprinting and 12 s of turning the pedals over slowly (between 20 and 30 rpm.) for a maximum of 60 repeats a session. Low-volume	Exercise: all out recovery: 20–30 rpm	15 weeks	20	3 times/week
Smith-Ryan et al. [44]	7	USA	35 males	Inactive	18–50	Active	High-intensity training (2 min-HIIT): 5 bouts of 2 min cycling with 1 min recovery utilizing undulating intensities (80–100% VO_2pea_k) or no exercise at all (CON). High-volume	HIIT intensity 80–100% of VO_2max_	3 weeks	20 min	3 times/week
Menz et al. [59]	7	Austria	8 females and 27 males	Well-trained athletes	25 ± 1	Active	Four 4-min interval bouts at an exercise intensity of 90–95% of the individual maximal heart rate (HRmax), separated by 4-min active recovery periods. High-volume	90–95% of HR_max_	4 weeks	32 min	3–4 session/week
Astorino et al. [60]	7	USA	192 women	Trained	22.5 ± 5.8	Active	** SIT (HIIT + SIT) consisted of 8–12 ‘‘all-out’’ sprints (4–6 min training duration per day) during which participants were required to pedal maximally High-volume interval training (HIIT + HIITHI) required repeated 2.5-min bouts of cycling with 60 s recovery HIIT + PER) consisted of 3 sessions of high-volume HIIT, 3 sessions of SIT, and 4 sessions of low-volume HIIT. High-volume	All out sprint	6 weeks	20–30 min	3 times/week
Czuba et al. [58]	7	Poland	16 males	Swimmers	19.1 ± 1.3	Active	Each training session consisted of a 10-min general warm-up, 45–55-min main part, and a 10-min cool-down. The main part of the circuit consisted of exercise performed on an upper limb rotator (50 W) with a cadence of 80 rpm lasting 60 s This circuit was repeated four times in the first four interval training sessions, after which a 5th circuit was added to increase the overall training load. High-volume	50–95% of VO_2max_	4 weeks	45–55 min	2 times/week
Rhibi et al. [5]	7	France	39 males	Physical education students	21.4 ± 1.1	Active	30 s IE run at 100–110% MAV alternating with 30 s at 50% MAV. High-volume	100–110% MAV	8 weeks	25 min (training and warm up, cool down)	3 times/week
Poon et al. [62]	7	Hong-kong	42 participants	Inactive	42 ± 5	Active	HIIT: 10 X 1-min bouts of running at 80–90% HRmax separated by 1-min active recovery. High-volume	80–90% HR_max_	8 weeks	21–29 min	3 times/week
Rhibi et al. [61]	8	France	37 males	Physical education students	21.9 ± 1.3	Active	30 s IE run at 100–110% MAV alternating with 30 s at 50% MAV. High-volume	100–110% MAV	8 weeks	25 min (training and warm up, cool down)	3 times/week

*MAV* Maximal aerobic velocity, *HIIT* High intensity interval training, *SIT* Sprint interval training, *IL-6* Interleukin-6, *TnF alpha* Tumor necrosis factor alpha, *BF* Body fat, *BMI* Body mass index, *CRP* C-reactive protein, *SSG* Small sided soccer game, *VO*_2max_ Maximal oxygen consumption, *HR* Heart rate, *rpm* Revolution per minute, *PER* Periodized interval training, *Row-HIIT* Traditional HIIT using rowing, *MM-HIIT* Multimodal HIIT, *RPE* Rating of perceived exertion, *SCT* Supramaximal cycling test, *VIFT* Maximum speed reached in the last stage of the 30–15 intermittent fitness test, *1 RM* one-repetition maximum, *IE* Intermittent exercise, *HIIRT* High-intensity interval resistance training

Thirteen studies (229 participants) examined the effects of interval training interspersed with passive recovery regimes on physical fitness and physiological performances in trained (6 studies) and untrained (7 studies) individuals. Eleven studies (501 participants) examined the effects of interval training interspersed with active recovery methods on measures of physical fitness and physiological parameters in trained (6 studies) and untrained individuals (5 studies) (Table 3).

**Table 3:** Physiotherapy Evidence Database (PEDro) score of the included longitudinal studies

Study	Eligibility criteria	Randomized allocation	Blinded allocation	Group homogeneity	Blinded subjects	Blinded therapists	Blinded assessor	Drop out\15%	Intention-to treat analysis	Between-group comparison	Point estimates and variability	PEDro sum
Martins et al. [45]	1	1	1	1	0	0	1	1	0	1	1	7
Trapp et al. [46]	1	1	1	1	0	0	1	1	0	1	1	7
Ben Abderrahman et al. [4]	1	1	1	1	0	0	1	1	0	1	1	7
Heydari et al. [63]	1	1	1	1	0	0	1	1	0	1	1	7
Buckley et al. [54]	1	1	1	1	0	0	1	1	0	1	1	7
Smith-Ryan et al. [44]	1	1	1	1	0	0	1	1	0	1	1	7
Menz et al. [59]	1	1	1	1	0	0	1	1	0	1	1	7
Kong et al. [53]	1	1	1	1	0	0	1	1	0	1	1	7
Astorino et al. [60]	1	1	1	1	0	0	1	1	0	1	1	7
Czuba et al. [58]	1	1	1	1	0	0	1	1	0	1	1	7
Aschendorf et al. [49]	1	1	1	1	0	0	1	1	0	1	1	7
Wiewelhove et al. [26]	1	1	1	1	0	0	1	1	0	1	1	7
Jabbour et al. [52]	1	0	1	1	0	0	1	1	0	1	1	6
Fransson et al. [47]	1	1	1	1	0	0	1	1	0	1	1	7
Menz et al. [50]	1	1	1	1	0	0	1	1	0	1	1	7
Rhibi et al. [5]	1	1	1	1	0	0	1	1	0	1	1	7
Evangelista et al. [55]	1	1	1	1	0	0	1	1	0	1	1	7
Alizadeh and Safarzade[51]	1	1	1	1	0	0	1	1	0	1	1	7
Moro et al. [56]	1	1	1	1	0	0	1	1	0	1	1	7
Poon et al. [62]	1	1	1	1	0	0	1	1	0	1	1	7
Arslan et al. [48]	1	0	1	1	0	0	1	1	0	1	1	6
Martínez-Rodríguez et al. [57]	1	1	1	1	0	0	1	1	0	1	1	7
Rhibi et al. [61]	1	1	1	1	0	0	1	1	0	1	1	8

The 24 studies used different interval training types (e.g., running, cycling, swimming) lasting between 3 and 15 weeks. The training duration mainly ranged between three [44] and 7 weeks [4], with two studies using a 12- and 15-week intervention period [45, 46].

### Effects of Interval Exercise Training Using Passive Recovery on Measures of Physical Fitness and Physiological Adaptations in Trained and Untrained Individuals

Table 4 summarizes the 13 studies that examined the effects of long-term interval training interspersed with passive recovery on measures of physical fitness and physiological parameters in both trained and untrained youth and adult individuals. Six studies [4, 26, 47–50] involved trained individuals and the seven remaining studies comprised untrained subjects [51–57]. Irrespective of the type of interval training or exercise protocol (type of exercise, duration, or intensity of exercise training), eleven out of 13 studies reported increases in measures of physical fitness (e.g., maximal aerobic velocity [MAV], Yo-Yo running test, jumping) and physiological parameters (e.g., VO_2max_, lactate threshold, blood pressures) in both trained (effect size for single studies: 0.13 < *d *< 3.27, small to very large) and untrained adults as well as trained youth (effect size: 0.17 < *d *< 4.19, small to very large).

**Table 4:** Effects of interval training using passive recovery on physical fitness and physiological adaptations in trained and untrained individuals

Study	Participants (number/age/sex)	Intervention	Physical fitness and physiological adaptations (unit)	Data		% Changes (*p* value)	Effect size (Cohen’s d)
				before	after		
*Trained individuals*							
Ben Abderrahman et al. [4]	24 male physical education students/20.4 ± 0.6	2 set x (8 rep x 30sIE) 100% MAV with 5 min recovery	VO_2max_ (ml.min^−1^.kg^−1^) MAV (km.h^−1^)	58.6 ± 4.2 15.5 ± 1.0	60.35 ± 4.7 16.5 ± 1.1	2.88 (*p *< 0.001) 6.4 (*p *< 0.001)	0.38 0.95
Fransson et al. [47]	39 elite male soccer players/21.1 ± 2.4	The drill was performed as a time trial drill in 30-s intervals separated by 150 s of passive recovery. The number of exercise intervals was six during the first intervention week, eight during the second and third weeks, and ten during the fourth week	Yo–Yo (m) Lactate (mmol.L^−1^)	222 ± 113 2.6 ± 1.9	323 ± 125 3.4 ± 1.7	45.49 (*p *< 0.05) 30.76 (*p *< 0.05)	0.85 0.44
Arslan et al. [48]	20 young male soccer players/14.2 ± 0.5	Intermittent running at 90–95% of players VIFT for 15 s (around the pitch), followed by 15 s of passive recovery	VO_2max_ (ml.min^−1^.kg^−1^) CMJ (cm) YYIRTL-1 (m)	46.8 ± 0.6 28.2 ± 2.0 1240 ± 75	48.9 ± 0.9 30.6 ± 1.8 1484 ± 74	4.4 (*p *≤ 0.05) 8.51 (*p *≤ 0.05) 19.6 (*p *≤ 0.05)	2.61 1.26 3.27
Aschendorf et al. [49]	24 females’ basketball players/15.1 ± 1.1	Two t types of HIIT sessions: Session A consisting of 4 × 4 HIIT with 3 min of recovery. Session B consisting of 2 × 30 s HIIT with 15 s recovery	YYIRTL-1 (m) CMJ (cm)	1498 ± 266 26.5 ± 3.3	1895 ± 421 27.0 ± 3.6	26.5 (*p *= 0.34) 1.8 (*p *= 0.10)	1.13 0.05
Wiewelhove et al. [26]	26 males intermittent sport (i.e., soccer, handball, basketball, hockey, floorball, tennis)/23.5 ± 2.5	Straight-line runs at 95–100%vV˙O2 with 2–4 min of rest	VO_2max_ (ml.min^−1^.kg^−1^) LT (mmol.L^−1^)	55.5 ± 4.2 12.5 ± 1.1	55.0 ± 3.6 12.8 ± 1.4	0.9 (*p *< 0.001) 0.024 (*p *< 0.05)	0.13 0.27
Menz et al. [50]	15 moderately trained healthy male and female/25.6 ± 2.6	Running HIIT (3–4 sets; 8 × 20 s, 10 s rest; set rest: 5 min)/functional HIIT executed with their own body weight (3–4 sets; 8 × 20 s, 10 s rest; set rest: 5 min)	VO_2max_ (ml.min^−1^.kg^−1^) HR_max_ (rpm)	47.8 ± 5.6 187 ± 10	54.1 ± 5.6 184 ± 10	13.17 (*p *= 0.011) 1.6 (*p *≤ 0.001)	1.13 0.42
Untrained individuals							
Alizadeh and Safarzade[51]	14 sedentary overweight male students/18.0 ± 1.5	30 s of all out exercise and 30 s of rest	Body weight (kg) BMI (kg.m^−2^) Body fat (%)	83.7 ± 3.6 27.8 ± 0.6 23.7 ± 3.0	80.4 ± 3.6 25.2 ± 0.7 20.3 ± 3.0	3.94 (*p *= 0.001) 9.35 (*p *= 0.002) 14.3 (*p *= 0.007)	0.87 3.99 0.33
Jabbour et al. [52]	30 sedentary healthy male and female/38	6 repetitions of SCT intervals with 2 min of passive recovery between each repetition. Each SCT repetition lasted 6 s, and participants were asked to pedal at maximal velocity against the resistance for 15 min	VO_2max_ (ml.min^−1^.kg^−1^) BMI (kg.m^−2^) Systolic BP (cmHg) Diastolic BP (cmHg) Hematocrit (%)	26.9 ± 1.4 30.1 ± 1.2 114.5 ± 2 78.3 ± 1.6 40.4 ± 3.1	27.2 ± 1.2 29.9 ± 1.2 109.1 ± 2.2 71.6 ± 1.6 39.9 ± 2.1	1.1 (*p *= 0.11) 0.66 (*p *= 0.11) 4.71 (*p *= < 0.001) 8.5 (*p *< 0.001) 1.2 (*p *= 0.13)	0.23 0.17 1.49 4.19 0.19
Kong et al. [53]	18 inactive overweight and obese females/19.8 ± 0.8	60 repetitions of high-intensity interval exercise (8 s cycling and 12 s passive recovery) on a cycle ergometer for 20 min	VO_2max_ (ml.min^−1^.kg^−1^) PPO (watts)	34.1 ± 5.7 134.2 ± 23.7	36.6 ± 6.6 150.2 ± 24.2	7.33 (*p *= 0.006) 11.9 (*p *< 0.001)	0.41 0.41
Buckley et al. [54]	28 recreationally active females/25.1 ± 5.6	Row-HIIT and MM-HIIT group performed 60 s of all-out intensity rowing followed by 3 min of rest (passive recovery) for a total of 6 rounds	VO_2max_ (ml.min^−1^.kg^−1^) AT (watts)	36.2 ± 5.7 27.2 ± 6.7	38.5 ± 5.4 29.7 ± 5.7	6.3 (*p *= 0.99) 9.1 (*p *= 0.09)	0.41 0.40
Evangelista et al. [55]	25 active healthy adults/28.7 ± 4.9	20 sets of 30 s all-out exercise and 30 s of passive recovery between sets	Horizontal jump (min) Push Ups	1.6 ± 0.3 27.8 ± 13.9	1.7 ± 0.3 34.3 ± 12.8	6.2 (*p *= 0.02) 23.38 (*p *= 0.02)	0.33 0.49
Moro et al. [56]	21 young healthy males and females/22.1 ± 1.9	HIIRT: first set of 6 repetitions at 80% 1RM followed by a 20″ rest; then, subjects were asked to lift the same weight until failure (habitually 2 or 3 repetitions) followed by another 20″ rest period with repetitions to fatigue	VO_2max_ (ml.min^−1^.kg^−1^) PPO (watts) Squat jump (sec)	41.4 ± 11.3 196.1 ± 52.4 0.32 ± 0.10	44.5 ± 9.1 213.0 ± 53.4 0.36 ± 0.08	7.3 (*p *> 0.05) 8.6 (*p *> 0.05) 12.5 (*p *= 0.02)	0.3 0.32 0.44
Martínez-Rodríguez et al. [57]	14 active, normal-weight females/27 ± 6	3 × 10 repetitions of 30 s of aerobic exercises all out (cycling, rowing, and running) interspersed by 30 s of rest (Passive recovery)	CMJ (cm)	17.5 ± 4.3	18.5 ± 3.9	5.7 (*p *< 0.001)	0.24

*CMJ* Countermovement jump, *LT* Lactate threshold, *PPO* Peak power output, *AT* Anaerobic threshold, *PVV* Plasma volume variation, *VT* Ventilatory threshold, *MAV* Maximal aerobic velocity-alpha, *BF* Body fat, *BMI* body mass index, *CRP* C-reactive protein, *SSG* Small sided soccer game, *VO*_2max_ Maximal oxygen consumption, *BP* Blood pressure, *HR* Heart rate, *YYIRTL-1 *Yo-Yo intermittent recovery test level 1, *Row-HIIT* Traditional HIIT using rowing, *MM-HIIT* Multimodal HIIT, *RPE* Rating of perceived exertion, SCT Supramaximal cycling test, *VIFT* Maximum speed reached in the last stage of the 30–15 intermittent fitness test, *1 RM* One-repetition maximum, *IE* Intermittent exercise, *HIIRT* High-intensity interval resistance training

Two studies were identified that examined the effects of passive recovery applied during interval training in young female basketball players aged 15.1 ± 1.1 years [49] and in male soccer players aged 14.2 ± 0.5 years. [48]. Both studies showed positive effects of passive recovery on VO_2max_, countermovement jump (CMJ), and the Yo-Yo intermittent running test level 1 (YYIRTL-1).

### Effects of Interval Exercise Training Using Active Recovery on Measures of Physical Fitness and Physiological Adaptations in Trained and Untrained Individuals

Table 5 summarizes the findings of eleven studies that examined the effects of interval training interspersed with active recovery on physical fitness and physiological parameters in trained and untrained adults. Six studies [4, 5, 58–61] involved trained individuals and the five remaining studies incorporated untrained subjects [44–46, 62, 63].

**Table 5:** Effects of interval exercise training using active recovery on physical fitness and physiological adaptations in trained and untrained individuals

Study	Participants (number/age/sex)	Intervention	Physical fitness and physiological adaptations	Data		% Changes (*p* value)	Effect size (Cohen’s d)
				before	after		
*Trained individuals*							
Ben Abderrahman et al. [4]	30 males’ physical education students/20.4 ± 0.6	2 set x (8 rep x 30sIE) 100% MAV with 5 min recovery	VO_2max_(ml.min^−1^.kg^−1^) MAV (km.h^−1^)	59.37 ± 7.5 16.1 ± 1.2	62.85 ± 7.9 17.0 ± 1.0	5.86 (*p *< 0.001) 5.55 (*p *< 0.001)	0.45 0.81
Czuba et al. [58]	16 male sprint swimmers/19.1 ± 1.3	Circuit based intermittent hypoxic training 2 times per week. Upper limb 60 s × 30 s rest. Lower limb 2 min × 3-min rest	VO_2max_ (ml.min^−1^.kg^−1^) Lactic acid (mmol.L^−1^) pH Change	56.0 ± 4.0 9.14 ± 0.92 0.147 ± 0.051	59.9 ± 4.3 11.05 ± 1.33 -0.178 ± 0.048	6.9 (*p *= 0.025) 20.8 (*p *= 0.001) 21 (*p *= 0.0129)	0.94 1.67 0.77
Menz et al. [59]	35 trained male and female athletes 25 ± 1	Four 4-min interval bouts at an exercise intensity of 90–95% of the individual maximal heart rate (HRmax), separated by 4-min active recovery periods	VO_2max_ (ml.min^−1^.kg^−1^)	47.8 ± 5.6	54.1 ± 5.6	13.1 (*p *= 0.011)	1.13
Astorino et al. [60]	192 trained women/21.9 T 1.9	SIT (HIIT + SIT) consisted of 8–12 ‘‘all-out’’ sprints (4–6 min training duration per day) during which participants were required to pedal maximally. High-volume interval training (HIIT + HIIT_HI_) required repeated 2.5-min bouts of cycling with 60 s recovery, leading to training duration equal to 12.5–17.5	VO_2max_ (ml.min^−1^.kg^−1^) VT a-vDO2	41.1 ± 4.9 112 ± 19 14.1 ± 1.6	44.6 ± 7.0 120 ± 18 14.3 ± 2.0	8.5 (*p *< 0.001) 7.1 (*p* 0.001) 1.41 (*p *< 0.001)	0.58 0.43 0.13
Rhibi et al. [5]	39 male physical education students/21.4 ± 1.1	30 s run at 100% MAV or 110% of MAV (EG110) alternating with 30 s active recovery	Hemoglobin (g/dl) Hematocrit (%) Lactate (mmol.L^−1^) PVV (%)	16.1 ± 0.8 49.3 ± 3.4 9.9 ± 2.5 − 9.0 ± 1.5	15.5 ± 0.6 47.1 ± 3.4 8.6 ± 1.9 − 6.8 ± 1.9	3.72 (*p *< 0.010) 4.46 (*p *= 0.085) 13.1 (*p *= 0.014) 22.2 (*p *= 0.014)	0.85 0.65 0.59 1.29
Rhibi et al. [61]	37 male physical education students/21.9 ± 1.3	30 s run at 100% MAV or 110% of MAV (EG110) alternating with 30 s active recovery (50% MAV)	MAV (km.h^−1^) Glucose (g/l) Insulin (μU.mL^−1^) Cortisol (ng.ml^−1^) IL-6 (pg.ml^−1^) TNF-α (pg.ml^−1^)	15.8 ± 1.6 6.4 ± 1.3 16.9 ± 1.7 386.0 ± 95.9 4.7 ± 1.2 6.8 ± 1.7	16.7 ± 1.5 5.6 ± 1.0 15.5 ± 1.7 465.7 ± 60.0 3.5 ± 1.1 5.2 ± 1.6	5.6 (*p *< 0.05) 12.5 (*p *= 0.021) 8.2 (*p *= 0.017) 20.4 (*p *< 0.001) 25.5 (*p *< 0.001) 23.5 (*p *< 0.001)	0.58 0.69 0.82 0.88 1.04 0.97
*Untrained individuals*							
Trapp et al. [46]	34 inactive healthy women/22.4 ± 0.7	Each subject performed 8 s of sprinting and 12 s of turning the pedals slowly (between 20 and 30 rpm) for a maximum of 60 repeats a session	VO_2max_ (ml.min^−1^.kg^−1^) HOMA-IR	28.8 ± 2.1 3.6 ± 0.7	36.4 ± 2.5 2.4 ± 0.7	23.38 (*p *< 0.05) 33.38 (*p *< 0.05)	3.29 1.71
Poon et al. [62]	24 physically inactive and overweight/obese Asian men/49.6 ± 7.8	HIIT: 10 X 1-min bouts of running at 80–90% HRmax separated by 1-min active recovery	VO_2max_ (ml.min^−1^.kg^−1^) BMI (kg.m^−2^) Systolic BP (cmHg) Diastolic BP (cmHg) Glucose (mmol/l)	32.5 ± 5.6 26.1 ± 1.6 120.6 ± 11.8 79.8 ± 6.8 4.90 ± 0.46	36.0 ± 6.2 25.8 ± 1.5 125.8 ± 8.8 75.2 ± 5.2 5.05 ± 0.29	10.7 *p *= 0.013 1.14 *p *= 0.035 3.6 (*p *= 0.377) 5.7 (*p *= 0.721) 3.06 (*p *= 0.656)	0.59 0.19 0.50 0.76 0.39
Martins et al. [45]	46 sedentary male and female obese individuals/34.4 ± 8.8	The HIIT protocol consisted of 8 s of sprinting and 12 s of recovery (pedals as slowly as possible)	VO_2max_ (ml.min^−1^.kg^−1^)	31.1 ± 4.9	33.9 ± 5.2	9 (*p *< 0.00)	0.55
Heydari et al. [63]	34 young overweight males/18–35	8 s sprint and 12 s of slow pedaling recovery for 20 min	VO_2max_ (ml.min^−1^.kg^−1^) SV Systolic BP (cmHg) Diastolic BP (cmHg)	33.9 ± 1.1 72 ± 4.2 120 ± 2.4 64 ± 1.8	39.8 ± 0.9 86 ± 5.1 115 ± 2.5 58 ± 1.8	17.4 (*p *< 0.0001) 19.4 (*p *< 0.0001) 4.1 (*p *= 0.004) 0.9 (*p *= 0.002)	5.87 3.00 2.04 3.33
Smith-Ryan et al. [44]	35 inactive overweight men/38.3 ± 11.5	5 bouts of 2 min cycling with 1 min recovery with passive recovery	Fat mass (kg) Lean mass (kg) Body fat (%) Glucose (mmol/l) Cholesterol (mg)	29.5 ± 0.9 69.5 ± 3.4 28.8 ± 1.4 98.9 ± 36.8 200.1 ± 48.4	28.3 ± 1.4 71.6 ± 2.4 27.5 ± 1.2 91.7 ± 16.3 175.3 ± 76.2	4.06 (*p *= 0.001) 3.2 (*p *= 0.001) 4.5 (*p *= 0.633) 7.2 (*p *= 0.008) 12.5 (*p *= 0.898)	0.95 0.76 1.03 0.29 0.36

*CMJ* Countermovement jump, *LT* Lactate threshold, *PPO* Peak power output, *AT* Anaerobic threshold, *a-v do2* Maximal arteriovenous difference, *HOMA _IR* Homeostasis model assessment of insulin sensitivity, *PVV* Plasma volume variation, *VT* Ventilatory threshold, *MAV* Maximal aerobic velocity, *IL-6* Interleukin-6, *TNF alpha* Tumor necrosis factor-alpha, *BF* Body fat, *BMI* Body mass index, *CRP* C-reactive protein, *SSG* Small sided soccer game, *VO*_*2max*_ Maximal oxygen consumption, *BP* Blood pressure, *HR* Heart rate, *YYIRTL-1* Yo-Yo intermittent recovery test level 1, *Row-HIIT* Traditional HIIT using rowing, *MM-HIIT* Multimodal HIIT, *RPE* Rating of perceived exertion, *SCT* Supramaximal cycling test, *VIFT* Maximum speed reached in the last stage of the 30–15 intermittent fitness test, *1 RM* One-repetition maximum, *IE* Intermittent exercise, *HIIRT* High-intensity interval resistance training

Irrespective of the type of interval training or exercise protocol (type of exercise, duration, or intensity of exercise training), nine out of 11 studies reported increases in physical fitness (e.g., MAV) and physiological parameters (e.g., VO_2max_, lactate threshold, blood pressures) in trained (effect size: 0.13 < *d *< 1.29, small to large) and untrained individuals (effect size: 0.19 < *d *< 3.29, small to very large).

## Discussion

Our main finding was that irrespective of the recovery type (passive or active) long-term interval training-induced enhancements in measures of physical fitness and physiological parameters in trained and untrained males and females aged 14–48 years.

### Effects of Interval Exercise Training Using Passive Recovery on Measures of Physical Fitness and Physiological Adaptations in Trained and Untrained Individuals

Our analysis showed that on average, exercise performance was increased after long-term interval exercise training with passive recovery in healthy-trained individuals. Nine studies [4, 26, 47–50, 58, 59, 63] were of high quality and included both sexes [4, 26, 47–50, 58, 59] and reported that passive recovery had a large positive effect on VO_2max_ and physical fitness using aerobic exercise training at 90–100% of VO_2max_. Eleven studies used aerobic interval training as an intervention [4, 26, 47–51, 53, 54, 56, 57], while another two studies used either sprint interval training [52] or repeated sprint ability as interventions [55]. Most included studies showed large effect sizes of passive recovery during long-term interval exercise training on VO_2max_. Two studies showed a small [26] or trivial [4] impact on VO_2max_. For jump performance, researchers from two studies reported a trivial [49] to large [48] effect after long-term interval exercise training with passive recovery on CMJ performance in youth male soccer [48] and youth female basketball players [49]. Results from these studies indicate positive effects of passive recovery on VO_2max_, CMJ, and the YYIRTL-1. This result has to be interpreted with caution due to the limited number of available studies. More research is needed on the effects of passive recovery on measures of physical fitness and physiological adaptations in youth.

Our analysis indicated small improvements in measures of physical fitness after long-term interval exercise training with passive recovery in healthy untrained individuals. Indeed, the seven included studies [51, 52, 54] were of high quality, included both sexes, and the population was restricted to healthy individuals [52, 54–57], overweight, and obese [51, 53] individuals, aged 18–38 years old. Three studies with passive recovery reported slight positive training-induced changes in VO_2max_ [52–54, 56], jump tests [55–57], muscle strength [55], and body composition [52]. Researchers from one study [51] reported a very large positive impact on the body mass index in overweight participants aged 18.0 ± 1.5 years. after a 30 s/30 s training program with a passive recovery.

The largest training-induced effects on VO_2max_ and physical fitness were observed in trained athletes compared to untrained individuals aged 18–38 years old. To better appreciate the impact of passive recovery on high-intensity interval exercise training, it is important to understand how this recovery mode relates to VO_2max_ and physical fitness. Accordingly, Ben Abderrahman et al. [4] showed that the longer time limit observed in trained adults could mainly be explained by the resynthesis of a higher proportion of the muscle phosphocreatine used during the 30 s intensive runs at 105% of MAV during the passive recovery. Another potential explanation for the observed result might be the difference in body mass between trained (74.2 ± 10.3 kg) and untrained individuals (67.0 ± 6.5 kg) [4].

The studies included in this systematic review reported that weight-bearing high-intensity interval exercises have a greater positive impact on anthropometrics [51] and cardiovascular [52] parameters compared with physical fitness and VO_2max_ in healthy non-obese or obese untrained individuals aged 18–38 years old.

Further, it was previously demonstrated that passive recovery facilitates a greater interval performance in trained athletes aged 20–25 years old [4, 47, 50]. The fact that athletes completed exercise training with large VO_2max_ increases and small changes in peak HR [50] and blood lactate [47] suggests that athletes could perform more bouts in this condition and, therefore, accumulate more time spent at high %VO_2max_ levels compared to untrained individuals. Regarding the effects of passive recovery during HIIT sessions on exercise performance, some studies [24, 38] indicated that, compared to active recovery, passive recovery was associated with a greater time to exhaustion (i.e., the accumulation of more work intervals or time spent at high intensities close to VO_2max_), but also a higher mean velocity/power development during work intervals when the number of bouts was fixed and the intensity self-regulated [24, 38].

### Effects of Long-Term Interval Exercise Training Using Active Recovery on Measures of Physical Fitness and Physiological Adaptations in Trained and Untrained Individuals

Our analysis showed that on average, exercise performance is slightly increased during long-term high interval exercise training with active recovery in healthy trained individuals. Indeed, six included studies [4, 5, 50, 58, 60, 61] were of high quality, included both sexes, and the population was restricted to young athletes aged 19–25 years. The studies, using aerobic interval training at 100–110% of MAV, found that active recovery had a small but positive effect on VO_2max_ [4, 60] and a large, positive effect on MAV [4, 5, 61]. Some other studies used sprint interval training [45, 46, 63], or maximal repeated sprint ability [5, 61] and observed significant small to large effects on VO_2max_.

Our analysis showed that on average, exercise performance can be improved (large to very large effects) after long-term interval exercise training with active recovery in healthy untrained individuals. Indeed, the four included studies [45, 46, 62, 63] were of high quality, including both sexes and restricted to healthy [46] and overweight/obese [45, 62, 63] subjects. Those studies found that active recovery had large to very large positive effects on VO_2max_ [45, 56, 62, 63] and was associated with a trivial change in body composition [62] using interval exercise training at 80–100% of power output or HR_max_. Three studies used maximal interval exercise training [45, 46, 63], and one study applied 80–90% HRmax during interval exercise training [62].

It is generally accepted that active recovery during long-term high-intensity interval exercise training has a very large effect on VO_2max_ in healthy or overweight untrained individuals compared to athletes [64]. In other words, these VO_2_ and mechanical efficiency data suggest that untrained individuals benefit more from maximal interval exercise training with active recovery than athletes [23].

The largest increase in VO_2max_ was reported in the study from Trapp et al., [46]. A possible explanation for this result might be related to the population and sex (inactive healthy females) with wide variations in VO_2max_ values. Moreover, the training duration was 15 weeks with 3 weekly sessions, which is a greater volume compared to the studies of Poon et al., [62] (3 times/week over 8 weeks) and Smith-Ryan et al., [44] (3 times per week over 3 weeks).

To the best of our knowledge, there is no study available that examined the effects of active recovery on physical fitness and physiological adaptations in youth.

### Study Limitations

There are some limitations to the current systematic review that should be noted. First, the studies examined were highly heterogeneous. In fact, the study populations varied in terms of sample size, sex, age (only two studies involved young individuals), and country of recruitment. Second, the training program's duration (3–15 weeks) and volume (15–45 min per session) were variable. Third, to our knowledge, no study currently available has reported the variation by effect size in physical fitness and physiological adaptations between trained and untrained individuals during high-intensity interval exercise training with passive or active recovery. Finally, due to the small number of studies included in our analysis, we were unable to consider sex and age as moderators of active recovery on physical exercise and VO_2max_. Furthermore, except for the work of Rhibi et al. [5], the majority of studies did not measure lactate concentration, which could provide more information on the relation between active recovery and lactate clearance during high-intensity exercise training.

### Practical Applications

The findings of our systematic review suggest that interval training, irrespective of the intensity level, has the potential to improve selected measures of physical fitness (e.g., MAV) and physiological responses (e.g., VO_2max_, blood pressure) similarly in trained and untrained adults and trained youth, regardless of the type of exercise and exercise dosage. More specifically, our findings suggest that the type of recovery (active or passive) applied during interval training results in similar training-induced outcomes, irrespective of the training status (trained, untrained) and sex (males, females). Thus, when long-term interval training programs (≥ 3 weeks) are performed, coaches and athletes can use either passive or active recovery modes. The decision should be based on the overall exercise programming parameters of the respective interval training. High exercise workloads may demand passive recovery whereas low workloads may favor active recovery.

## Conclusions

To conclude, our findings suggest that irrespective of the recovery mode (passive or active), long-term interval exercise training has the potential to enhance physical fitness and physiological adaptations in trained and untrained male and female adults. More research examining the effects of passive or active recovery on measures of physical fitness and physiological adaptations in youth is recommended.

## Data Availability

All data supporting the findings of this study are available in this published article.

## Abbreviations

1 RM: One-repetition maximum
AT: Anaerobic threshold
a-v do2: Maximal arteriovenous difference
BF: Body fat
BMI: Body mass index
CMJ: Countermovement jump
CRP: C-reactive protein
ES: Effect size
FITT: Frequency, intensity, time, and type
HII_RT_: High-intensity interval resistance training
HIIT: High-intensity interval training
HOMA-IR: Homeostasis model assessment of insulin sensitivity
HR: Heart rate
IE: Intermittent exercise
IL-6: Interleukin-6
LT: Lactate threshold
MAV: Maximal aerobic velocity
MM-HIIT: Multimodal HIIT
PEDro: Physiotherapy Evidence Database
PER: Periodized interval training
PGC-1α mRNA: Peroxisome proliferator-activated receptor-γ coactivator-1α
PPO: Peak power output
PRISMA: Preferred Reporting Items for Systematic Reviews and Meta-Analyses
PVV: Plasma volume variation
Row-HIIT: Traditional HIIT using rowing
RPE: Rating of perceived exertion
Rpm: Revolution per minute
SCT: Supramaximal cycling test
SIT: Sprint interval training
SSG: Small side soccer game
TnF alpha: Tumor necrosis factor-alpha
VIFT: Maximum speed reached in the last stage of the 30–15 intermittent fitness test
VO_2max_: Maximal oxygen uptake
VT: Ventilatory threshold
YYIRTL-1: Yo-Yo intermittent running test level 1

## References

[1] Pollock ML, Jackson AS, Pate RR (1980). Discriminant analysis of physiological differences between good and elite distance runners. Res Q Exerc Sport.

[2] Brandon LJ (1995). Physiological factors associated with middle distance running performance. Sports Med.

[3] Midgley AW, McNaughton LR, Wilkinson M (2006). Is there an optimal training intensity for enhancing the maximal oxygen uptake of distance runners?. Sports Med.

[4] Ben Abderrahman A, Zouhal H, Chamari K, Thevenet D, de Mullenheim P-Y, Gastinger S (2013). Effects of recovery mode (active vs passive) on performance during a short high-intensity interval training program: a longitudinal study. Eur J Appl Physiol.

[5] Rhibi F. Adaptations physiologiques à l’exercice intermittent court et chronique: Rennes 2; 2019.

[6] Billat VL, Flechet B, Petit B, Muriaux G, Koralsztein J-P (1999). Interval training at V˙O2max: effects on aerobic performance and overtraining markers. Med Sci Sports Exer.

[7] Baar K (2006). To perform your best: work hard not long. J Physiol.

[8] Egan B, Zierath JR (2013). Exercise metabolism and the molecular regulation of skeletal muscle adaptation. Cell Metab.

[9] Bird SR, Linden M, Hawley JA (2014). Acute changes to biomarkers as a consequence of prolonged strenuous running. Ann Clin Biochem.

[10] Buchheit M, Laursen PB (2013). High-intensity interval training, solutions to the programming puzzle. Sports Med.

[11] Buchheit M, Laursen P (2013). Science and application of high-intensity interval training: solutions to the programming puzzle. Sports Med.

[12] Seiler S (2010). What is best practice for training intensity and duration distribution in endurance athletes?. Int J Sports Physiol Perform.

[13] Tschakert G, Hofmann P (2013). High-intensity intermittent exercise: methodological and physiological aspects. Int J Sports Physiol Perform.

[14] Wenger HA, Bell GJ (1986). The interactions of intensity, frequency and duration of exercise training in altering cardiorespiratory fitness. Sports Med.

[15] Eddy DO, Sparks KL, Adelizi DA (1977). The effects of continuous and interval training in women and men. Eur J Appl Physiol.

[16] Gorostiaga EM, Walter CB, Foster C, Hickson RC (1991). Uniqueness of interval and continuous training at the same maintained exercise intensity. Eur J Appl Physiol.

[17] Tabata I, Nishimura K, Kouzaki M, Hirai Y, Ogita F, Miyachi M (1996). Effects of moderate-intensity endurance and high-intensity intermittent training on anaerobic capacity and VO~ 2~ m~ a~ x. Med Sci Sports Exerc.

[18] Franch J, Madsen K, Djurhuus MS, Pedersen PK (1998). Improved running economy following intensified training correlates with reduced ventilatory demands. Med Sci Sports Exerc.

[19] Cicioni-Kolsky D, Lorenzen C, Williams MD, Kemp JG (2013). Endurance and sprint benefits of high-intensity and supramaximal interval training. Eur J Sport Sci.

[20] Milanović Z, Sporiš G, Weston M (2015). Effectiveness of high-intensity interval training (hit) and continuous endurance training for VO_2max_ improvements: a systematic review and meta-analysis of controlled trials. Sports Med.

[21] Sloth M, Sloth D, Overgaard K, Dalgas U (2013). Effects of sprint interval training on VO_2max_ and aerobic exercise performance: a systematic review and meta-analysis. Scand J Med Sci Sports.

[22] Hottenrott L, Möhle M, Ide A, Ketelhut S, Stoll O, Hottenrott K (2021). Recovery from different high-intensity interval training protocols: comparing well-trained women and men. Sports.

[23] Zouhal H, Hammami A, Tijani JM, Jayavel A, de Sousa M, Krustrup P (2020). Effects of small-sided soccer games on physical fitness, physiological responses, and health indices in untrained individuals and clinical populations: a systematic review. Sports Med.

[24] Coates AM, Joyner MJ, Little JP (2023). A perspective on high-intensity interval training for performance and health. Sports Med.

[25] Iaia FM, Ermanno R, Bangsbo J (2009). High-intensity training in football. Int J Sports Physiol Perform.

[26] Wiewelhove T, Schneider C, Schmidt A, Döweling A, Meyer T, Kellmann M (2018). Active recovery after high-intensity interval-training does not attenuate training adaptation. Front Physiol.

[27] Wahl B, Reichmann D, Niks D, Krompholz N, Havemeyer A, Clement B (2010). Biochemical and spectroscopic characterization of the human mitochondrial amidoxime reducing components hmARC-1 and hmARC-2 suggests the existence of a new molybdenum enzyme family in eukaryotes*. J Biol Chem.

[28] MacInnis MJ, Zacharewicz E, Martin BJ, Haikalis ME, Skelly LE, Tarnopolsky MA (2017). Superior mitochondrial adaptations in human skeletal muscle after interval compared to continuous single-leg cycling matched for total work. J Physiol.

[29] Kellmann M, Bertollo M, Bosquet L, Brink M, Coutts AJ, Duffield R (2018). Recovery and performance in sport: consensus statement. Int J Sports Physiol Perform.

[30] Mujika I (2017). Quantification of training and competition loads in endurance sports: methods and applications. Int J Sport Physiol Perfor.

[31] Hebestreit H, Mimura KI, Bar-Or O (1993). Recovery of muscle power after high-intensity short-term exercise: comparing boys and men. J Appl Physiol.

[32] Dorado C, Sanchis-Moysi J, Calbet JAL (2004). Effects of recovery mode on performance, O_2_ uptake, and O_2_ deficit during high-intensity intermittent exercise. Can J Appl Physiol.

[33] Thiriet M, Graham JMR, Issa RI (1993). A computational model of wall shear and residence time of particles conveyed by steady flow in a curved tube. J Phys III.

[34] Weltman A, Stamford BA, Moffatt RJ, Katch VL (1977). Exercise recovery, lactate removal, and subsequent high intensity exercise performance. Res Quart Am All Health Phys Educ Recreat.

[35] Bangsbo J, Graham T, Johansen L, Saltin B (1994). Muscle lactate metabolism in recovery from intense exhaustive exercise: impact of light exercise. J Appl Physiol.

[36] Dupont G, Blondel N, Berthoin S (2003). Performance for short intermittent runs: active recovery vs. passive recovery. Eur J Appl Physiol.

[37] Madueno MC, Guy JH, Dalbo VJ, Scanlan AT (2019). A systematic review examining the physiological, perceptual, and performance effects of active and passive recovery modes applied between repeated-sprints. J Sports Med Phys Fitness.

[38] Perrier-Melo RJ, D'Amorim I, Meireles Santos T, Caldas Costa E, Rodrigues Barbosa R, Costa MD (2021). Effect of active versus passive recovery on performance-related outcome during high-intensity interval exercise. J Sports Med Phys Fitness.

[39] Moher D, Liberati A, Tetzlaff J, Altman DG (2009). Preferred reporting items for systematic reviews and meta-analyses: the PRISMA statement. Ann Intern Med.

[40] McKay AKA, Stellingwerff T, Smith ES, Martin DT, Mujika I, Goosey-Tolfrey VL (2022). Defining training and performance caliber: a participant classification framework. Int J Sports Physiol Perform.

[41] Maher CG, Sherrington C, Herbert RD, Moseley AM, Elkins M (2003). Reliability of the PEDro scale for rating quality of randomized controlled trials. Phys Ther.

[42] Cohen J (1988). Statistical power analysis for the behavioral sciences.

[43] Hopkins WG, Marshall SW, Batterham AM, Hanin J (2009). Progressive statistics for studies in sports medicine and exercise science. Med Sci Sports Exerc.

[44] Smith-Ryan AE, Melvin MN, Wingfield HL (2015). High-intensity interval training: modulating interval duration in overweight/obese men. Phys Sportsmed.

[45] Martins C, Kazakova I, Ludviksen M, Mehus I, Wisloff U, Kulseng B (2016). High-intensity interval training and isocaloric moderate-intensity continuous training result in similar improvements in body composition and fitness in obese individuals. Int J Sport Nutr Exerc Metab.

[46] Trapp EG, Chisholm DJ, Freund J, Boutcher SH (2008). The effects of high-intensity intermittent exercise training on fat loss and fasting insulin levels of young women. Int J Obes.

[47] Fransson D. Game demands and fatigue profiles in elite football–an individual approach-Implications for training and recovery strategies. 2019.

[48] Arslan E, Orer G, Clemente F (2020). Running-based high-intensity interval training vs small-sided game training programs: effects on the physical performance, psychophysiological responses and technical skills in young soccer players. Biol Sport.

[49] Aschendorf PF, Zinner C, Delextrat A, Engelmeyer E, Mester J (2019). Effects of basketball-specific high-intensity interval training on aerobic performance and physical capacities in youth female basketball players. Phys Sportsmed.

[50] Menz V, Marterer N, Amin SB, Faulhaber M, Hansen AB, Lawley JS (2019). Functional vs. running low-volume high-intensity interval training: effects on VO_(2)max_ and muscular endurance. J Sports Sci Med.

[51] Alizadeh H, Safarzade A (2019). Effect of a 6-week running sprint interval training protocol on serum meteorin-like hormone, insulin resistance, and body composition in overweight adolescents. Med Sport.

[52] Jabbour G, Iancu H-D, Zouhal H, Mauriège P, Joanisse DR, Martin LJ (2018). High-intensity interval training improves acute plasma volume responses to exercise that is age dependent. Physiol Rep.

[53] Kong Z, Fan X, Sun S, Song L, Shi Q, Nie J (2016). Comparison of high-intensity interval training and moderate-to-vigorous continuous training for cardiometabolic health and exercise enjoyment in obese young women: a randomized controlled trial. PLoS ONE.

[54] Buckley S, Knapp K, Lackie A, Lewry C, Horvey K, Benko C (2015). Multimodal high-intensity interval training increases muscle function and metabolic performance in females. Appl Physiol Nutr Metab.

[55] Evangelista AL, La Scala TC, Machado AF, Pereira PE, Rica RL, Bocalini DS (2019). Effects of a short-term of whole-body, high-intensity, intermittent training program on morphofunctional parameters. J Bodyw Mov Ther.

[56] Moro T, Marcolin G, Bianco A, Bolzetta F, Berton L, Sergi G (2020). Effects of 6 weeks of traditional resistance training or high intensity interval resistance training on body composition, aerobic power and strength in healthy young subjects: a randomized parallel trial. Int J Environ Res Pub Health.

[57] Martínez-Rodríguez A, Rubio-Arias JA, García-De Frutos JM, Vicente-Martínez M, Gunnarsson TP (2021). Effect of high-intensity interval training and intermittent fasting on body composition and physical performance in active women. Int J Environ Res Public Health.

[58] Czuba M, Wilk R, Karpiński J, Chalimoniuk M, Zajac A, Langfort J (2017). Intermittent hypoxic training improves anaerobic performance in competitive swimmers when implemented into a direct competition mesocycle. PLoS ONE.

[59] Menz V, Strobl J, Faulhaber M, Gatterer H, Burtscher M (2015). Effect of 3-week high-intensity interval training on VO_2max_, total haemoglobin mass, plasma and blood volume in well-trained athletes. Eur J Appl Physiol.

[60] Astorino TA, Edmunds RM, Clark A, King L, Gallant RA, Namm S (2017). High-intensity interval training increases cardiac output and VO_2max_. Med Sci Sports Exerc.

[61] Rhibi F, Dhahbi W, Jebabli N, Bideau B, Prioux J, Attia MB (2022). Optimization of high-intensity-interval-training program intensity to improve aerobic performance in healthy active subjects. Med Sport.

[62] Poon ET-C, Siu PM-F, Wongpipit W, Gibala M, Wong SHS (2022). Alternating high-intensity interval training and continuous training is efficacious in improving cardiometabolic health in obese middle-aged men. J Exerc Sci Fitness.

[63] Heydari M, Freund J, Boutcher SH. The effect of high-intensity intermittent exercise on body composition of overweight young males. J Obes. 2012;2012. 10.1155/2012/480467 PMC3375095 22720138

[64] Zouhal H, Ben Abderrahman A, Khodamoradi A, Saeidi A, Jayavel A, Hackney AC (2020). Effects of physical training on anthropometrics, physical and physiological capacities in individuals with obesity: a systematic review. Obes Rev.

